# Work stress and alcohol consumption among adolescents: moderation by family and peer influences

**DOI:** 10.1186/1471-2458-14-1303

**Published:** 2014-12-18

**Authors:** Xianfang C Liu, Katherine M Keyes, Guohua Li

**Affiliations:** Department of Community Health Sciences, University of Calgary, TRW Building, 3280 Hospital Drive, Calgary, NW T2N 4Z6 Canada; Department of Epidemiology, Columbia University Mailman School of Public Health, New York, NY 10032 USA

**Keywords:** Adolescent employment, Alcohol use, Work stress

## Abstract

**Background:**

Excessive alcohol use in adolescence can be detrimental to health and academic performance. Few studies consider the moderating effects of parental and peer influence within the context of adolescent work outside of the school environment. This study aims to examine work stress among adolescents and the association with alcohol use and drunkenness, in the context of parental and peer influences.

**Methods:**

Grade 12 students who participated in Monitoring the Future surveys between 2005 and 2009 (n = 12,341) were included in this study. Independent variables included work stress (job satisfaction, perceived safety, and perceived safety of possessions), self-reported perceptions towards academics and influence from parents and peers. Frequency of alcohol use and drunkenness were measured for lifetime, last 30 days and 12 months. The moderating effects of academic aspiration, parental, and peer influence were assessed on the relationship between work stress and alcohol use.

**Results:**

Any work stress was positively associated with alcohol use over the past 12 months (odds ratio = 1.12, 95% confidence interval (CI) 1.02-1.23). Stratified analysis found that peer influence significantly moderated the relationship between work stress and alcohol use over the lifetime and past 12 months. Among adolescents with work stress, odds ratios of alcohol use over the lifetime was 0.83 (95% CI 0.71-0.97) for those with low negative peer influence and 1.09 (95% CI 0.97-1.22) for those with high negative peer influence.

**Conclusions:**

Problematic drinking patterns were more apparent among high school students who experienced stress at work. Positive peer influence, however, may buffer the adverse effect of work stress on alcohol use.

**Electronic supplementary material:**

The online version of this article (doi:10.1186/1471-2458-14-1303) contains supplementary material, which is available to authorized users.

## Background

Alcohol use in adolescence is associated with a number of adverse consequences, including depression and suicide ideation [1, 2]; motor vehicle crashes [3]; and alcohol disorders [4]. Alcohol use in adolescence also predicts alcohol use trajectories over the life course [5] and alcohol dependence in adulthood [6]. Delaying the onset of alcohol use in adolescence is an important public health issue [7].

Adolescent alcohol use has been considered a social behavior and thus dependent on social influences experienced in one’s life, such as stress [8]. Indeed, social scientists conceptualized that stressful life events gave rise to chronic strains [9], which may influence risk of alcohol use [10–12]. Research has demonstrated that the stress process was also strongly associated with alcohol use in adolescence [10–12] and influenced individuals experiences, which involved levels of social structures, social institutions, and interpersonal interactions [9].

Working adolescents may experience factors in the work environment that create stress. There are two prevailing job stress models: the job strain model and the effort-reward imbalance model [13]. The job strain model posits that job strain occurs as a result of high job demands and low job control. The effort-reward imbalance model posits that there is a trade-off between the effort put into work and the reward gained, such as money, esteem, and job status [14]. These models have been tested in the past predominately to explain the role of work stress and adverse health effects, and have been associated with heart disease [13]. To date, there has been no job stress model that explored the relationship between adolescent job stress and alcohol use.

Work stress can be influenced by situations in the workplace or the perception of the workplace [15]. For example, a potential work stressor would be long hours worked, which has been established as a robust risk factor for early onset alcohol use during adolescence [16–19]. Furthermore, stress caused by problems at work may be an underlying factor for increased alcohol consumption [20]. Other forms of work stress could be attributed to physical or emotional stressors from the workplace, such as excess work, lack of autonomy, and poor working environment [17]. In addition, job satisfaction and job involvement was positively associated to job stressors and alcohol use [11, 21].

Adolescent academic aspiration and relationships with parents and peers are social structures that may underlie the association between adolescent work stress and alcohol use. First, academic aspiration may influence the relation between work stress and alcohol use among adolescents; specifically, adolescents with low engagement in school and low academic aspirations were more likely to engage in work outside of school, which in turn, increased their risk of alcohol use [22, 23]. Long hours (>15 hours per week) worked by adolescents was negatively correlated with grade point average (a measure of scholastic achievement) and educational attainment [23]. Second, parental and peer alcohol use was strongly predictive of adolescent alcohol use [17, 19, 24–27]. For example, frequent parent–child discussion about alcohol use norms promoted abstinence-based alcohol use which increased the likelihood that children viewed alcohol use as unacceptable [17]. Parent alcohol use influenced what they discussed with their children and in some cases it may have increased adolescent alcohol use [19]. Peer influence from close friends and friendship groups were found to influence initiation of alcohol use in adolescents [25]. However, the context where peers influenced the adolescent may have depended on how they were selected [25]. For example, adolescents may have used alcohol in order to fit in with their friends who also use alcohol, and conversely, adolescents who do not use alcohol may have rejected friendship with peers who do [25]. In addition to individual peer drunkenness, school-level peer drunkenness was positively associated with adolescent lifetime drunkenness [28]. Adolescents with low parental supervision and/or parent-adolescent relationship [29, 30], and deviant peer relations [31], were at higher risk for alcohol use compared to other adolescents. Conversely, social support (in the form of providing psychological and material resources) from family members and peers was shown to provide an overall beneficial effect (direct-effect model), as well as protecting those that experience stressful events (buffering model) [31]. Parent and peer relationships may provide an indication of the extent to which these variables moderate adolescent work stress.

In the present study, we examined the effect that work stress has on adolescent alcohol use and drunkenness in the context of academic aspiration and influences from parents and peers. We utilized nationally-representative information on school-attending adolescents in the 12th grade to examine the association of work stress with the frequency of alcohol use and drunkenness in adolescents, while considering possible confounding or moderating effects of parent and peer influence, and academic aspiration.

## Methods

### Sample

This study was approved by the Institutional Review Boards at University of Michigan and Columbia University. This study consisted of grade 12 participants from the annual Monitoring the Future (MTF) national survey. We analyzed data over five years from 2005 to 2009. The methods are described elsewhere [32, 33]. MTF is an annually conducted cross-sectional nationally representative survey of high-school attending adolescents. Each survey included about 15,000 respondents from over 400 public and private secondary schools. MTF uses a multi-stage random sampling method to achieve a representative sample of American secondary school students in the 48 contiguous states. All schools were asked to participate for two years to ensure that trends were captured. 79% of all sampled grade 12 participants completed questionnaires in 2008 [32] and 82% completed questionnaires in 2009 [33]. The total analytic sample size was 12,341, which captured adolescents that responded to questions about work stress.

### Measures

#### Alcohol use and drunkenness frequency

The survey asked: “On how many occasions have you had alcoholic beverages to drink-more than just a few sips in your lifetime? During the last 12 months? During the last 30 days?”, and “On how many occasions have you been drunk or very high from drinking alcoholic beverages in your lifetime? During the last 12 months? During the last 30 days?” Alcohol use over the lifetime and past 12 month was categorized as 0 times, 1–19, and 20+; alcohol use over the past 30 days was categorized as 0 times, 1–5, and 6+. Drunkenness over the lifetime, 12 months, and past 30 days were categorized as 0 or 1+ times.

#### Work stress

This variable measured perceived factors in the work environment that could contribute to stress. Work stress was assessed by three questions: “How satisfied are you with your job?” “How satisfied are you with your personal safety on your job?” “The safety of things you own from being stolen or destroyed on your job?” These questions measured the perceived job safety, perceived personal safety at work, and the perceived safety of one’s possessions at work. The responses to these three questions were coded as 0 or 1 to represent satisfied or dissatisfied. These values were aggregated to create the work stress variable, and thus it was an ordinal variable, which ranged from 0 to 3. We further dichotomized the work stress variable so that adolescents either had ‘no work stress’ or ‘any work stress’. Adolescents who reported that they were dissatisfied to any of the questions were classified as having any work stress (33.71%). Adolescents that reported being satisfied to all of the questions were classified as having no work stress (66.29%).

#### Academic aspiration

This variable measured the perceived importance of academics by determining how frequently students attended school and participated in activities. Questions included: “How do you feel about going to school?”, “How often did you enjoy being in school?”, “About how many hours do you spend in an average week on all of your homework including both in school and out of school?”, “How often do you feel that the school work you are assigned is meaningful and important?”, “Over the past year, how often did you try to do your best work in school?” All responses were aggregated to create the academic aspiration variable, which ranged from 0 to 8. Since the distribution of this variable was skewed and there was a linear relationship with alcohol use and drunkenness, we dichotomized academic aspiration into ‘low academic aspiration’ and ‘high academic aspiration’. 47.79% of adolescents had low academic aspiration while 52.21% had high academic aspiration.

#### Parent influences

Positive parent influence measured closeness to parents and parental support and was assessed based on the questions: “Over the last 12 months, how often have you argued or had a fight with either of your parents?”, “How important is living close to parents and relatives?”, “How often do your parents check on whether you have done your homework? Provide help with your homework when it’s needed?” These responses were rated as never/rarely or sometimes/often. Positive parent influence included responses that showed the adolescent agreed and depended on their parents, or received support from them were given a value of 1, otherwise a 0. The responses were aggregated and created the parental influence variable, which ranged from 0 to 2. The distribution of this variable was skewed and thus we dichotomized the variable based on the distribution of values. 73.33% of adolescents experienced positive parental influence, while 26.67% experienced none or low positive parent influence.

#### Peer influences

This variable measured negative peer influences such as peer alcohol use and alcohol use encouragement. Questions included: “How important is having strong friendships to you in your life?” Other questions asked about their alcohol use influences from friends: “How many friends would you estimate drink alcoholic beverages? Get drunk at least once a week?” and “How would your close friends feel if you take one or two drinks nearly every day? Four or five drinks nearly every day? Five or more drinks once or twice every weekend?” If responses indicated that friends were important and they encouraged alcohol use then a value of 1 was assigned, otherwise a 0. The peer influence variable ranged from 0 to 5. Since the distribution was skewed and there was a linear association between peer influence and alcohol use, the variable was dichotomized. 44.07% of adolescents experienced none or low negative peer influence, and 55.93% experienced high negative peer influence.

#### Control variables

General life satisfaction, average grade in school, father and mother education levels, gender, race/ethnicity, and region of residence could potentially confound the relationship between work stress and academic aspiration [23, 34–36] and were therefore controlled in all analyses. General life satisfaction could be correlated with job satisfaction depending on the strength of the influence. Average grade in school and parental education have been shown to be correlated with hours worked [23]. Similarly, gender, race/ethnicity, and region of residence were correlated with academic performance and school grades [34] as well as work stress [35, 36].

Control variables were determined by single questions that asked participants to rank their general life satisfaction, average grade in school, gender, race/ethnicity, region of residence, and parental education.

### Statistical analysis

The variables work stress, academic aspiration, parental influence, and peer influence were assessed based on questions in the MTF survey that are described above. Table 1 shows the distribution of adolescents for each variable and the population characteristics. We adjusted for multiple comparisons by applying the Bonferroni correction to the chi square tests.

**Table 1:** **The proportion of explanatory and demographic variables stratified by low or high work stress**

Variables	% of participants (n = 12,341)	% with no work stress (n = 8181)	% with any work stress (n = 4160)	Adjusted p-value
**Any work stress**	33.71	--	--	--
Academic aspiration (high)*	52.21	57.04	42.72	<0.0001
Parent influence (discourage alcohol use)*	73.33	77.01	66.09	<0.0001
Peer influence (encourage alcohol use)*	55.93	56.50	54.81	0.074
**General life satisfaction (yes)**	87.19	90.31	81.05	<0.0001
**Average grade (A)**	34.72	36.18	31.76	<0.0001
**Average grade (B)**	48.01	48.12	47.77	<0.0001
**Gender (male)**	47.63	49.12	44.61	<0.0001
**Race (white)**	71.30	74.34	65.08	<0.0001
**Race (black)**	12.48	11.04	15.41	<0.0001
**Region (rural)**	41.56	42.11	40.40	<0.096
**Father education level (college)**	39.27	42.88	31.71	<0.0001
**Mother education level (college)**	41.55	44.67	35.16	<0.0001

*Cutpoints for academic aspiration was 0 to 5 for low aspiration, 6 to 8 was high aspiration. For parental influence, cut offs were 0 to 1 for negative influence and 2 for positive influence. 0 to 3 was positive peer influence and 4 to 5 was negative peer influence.

We conducted univariate analysis to test individual associations between work stress, academic aspirations, parental influence, and peer influence with alcohol use and drunkenness. We conducted stratified logistic regression to analyze whether the association between work stress and alcohol use/drunkenness was moderated by academic aspiration, parental influence, and peer influence. To test the significance of the interaction, we conducted multinomial logistic regression separately for each moderator.

As a sensitivity analysis, we conducted Poisson analysis to model the effect of alcohol use and drunkenness frequency as ordinal outcomes. We examined the effect of work stress, peer influence, academic aspiration, and parental influence variables before they were dichotomized. Work stress had four levels, peer influence had six levels, academic aspiration nine levels, and parental influence three levels.

All analyses were conducted with SAS statistical software (Cary, NC) to obtain ORs and 95% confidence intervals.

## Results

Table 1 shows the proportion of adolescents that reported the explanatory and demographic variables stratified by work stress and their significance. There were a total of 12,341 participants included in this analysis. The p-values were adjusted for multiple comparisons using Bonferroni correction.

Table 2 shows the associations between work stress, academic aspiration, parental influence, and peer influence with alcohol use and drunkenness. Work stress was significantly associated with alcohol use over the past 12 months (OR = 1.12 (1.02-1.23)). Academic aspiration, parent, and peer influence were linearly associated with alcohol use and drunkenness. High academic aspiration and positive parent influence were negatively associated with alcohol use and drunkenness, while negative peer influence increased alcohol use and drunkenness. High negative peer influence greatly increased alcohol used over the past 12 months (OR = 6.66 (6.00 -7.38)).

**Table 2:** **Association between work stress, academic aspirations, parental and peer influences on alcohol use**

	Alcohol use			Drunkenness		
	Lifetime	12 months	30 days	Lifetime	12 months	30 days
	OR (95% CI)	OR (95% CI)	OR (95% CI)	OR (95% CI)	OR (95% CI)	OR (95% CI)
Any work stress*	1.04 (0.95-1.14)	1.12 (1.02-1.23)	1.06 (0.96-1.16)	1.07 (0.97-1.19)	1.07 (0.96-1.20)	1.12 (0.96-1.30)
High academic aspiration*	0.50 (0.45-0.54)	0.48 (0.44 -0.53)	0.53 (0.48-0.58)	0.46 (0.41-0.50)	0.39 (0.35-0.44)	0.39 (0.34-0.45)
Positive parental influence*	0.86 (0.78-1.11)	0.85 (0.77-0.94)	0.91 (0.83-1.01)	0.85 (0.76-0.95)	0.79 (0.70-0.89)	0.83 (0.71-0.98)
Negative peer influence*	5.52 (5.00 -6.09)	6.66 (6.00 -7.38)	6.06 (5.44 -6.74)	6.82 (6.12 -7.60)	12.75 (10.93 -14.88)	10.83 (8.44 -13.90)

*controlling for life satisfaction, GPA, gender, race, region, and parental education.

Table 3 shows the relationship between work stress and alcohol use or drunkenness after stratification by peer influence. Peer influence moderated the relationship between work stress and alcohol use over the lifetime and past 12 months (Table 3). Among adolescents with any work stress and high negative peer influences, odds ratio of alcohol use over the lifetime was 1.09 (0.97-1.22). Among those with work stress and low negative peer influence, the odds ratio of alcohol use over the lifetime was 0.83 (0.71-0.97); chi-square = 6.56, p = 0.01. Peer influence did not significant moderate the relationship between work stress and drunkenness.

**Table 3:** **Adjusted odds ratio of alcohol use and drunkenness among adolescents with work stress, stratified by peer influence**

	Alcohol use			Drunkenness		
	Lifetime	12 months	30 days	Lifetime	12 months	30 days
	OR (95% CI)	OR (95% CI)	OR (95% CI)	OR (95% CI)	OR (95% CI)	OR (95% CI)
Any work stress and high negative peer influence*	1.09 (0.97-1.22)	0.98 (0.87 (1.11)	1.02 (0.91-1.14)	1.06 (0.092-1.22)	1.03 (0.89-1.19)	0.94 (0.80-1.12)
Any work stress and low negative peer influences*	0.83 (0.71-0.97)	0.84 (0.71-0.98)	0.87 (0.72-1.06)	0.88 (0.73-1.05)	0.85 (0.63-1.14)	0.92 (0.55-1.54)
Interaction between any work stress and peer influence* Chi-square (p-value)	6.56 (0.010)	6.10 (0.014)	3.54 (0.060)	2.81 (0.094)	1.60 (0.21)	0.43 (0.513)

*The reference group was adolescents with no work stress. Control variables include life satisfaction, GPA, gender, race, region, and parental education.

Parameter estimates from Poisson analysis showed that work stress, peer influence, and academic aspiration were associated with alcohol use and drunkenness (Additional file 1). There were stepwise increases or decreases in the parameter estimates as work stress, negative peer influence, and positive academic aspirations increased. The parameter estimates confirmed that were was a linear association between work stress, peer influence, and academic aspiration with alcohol use and drunkenness. Type 1 analysis also found that these models were statistically significant, p-value < 0.05 (not shown). There was no significant difference in alcohol use or drunkenness between different levels of parental influence. The results from Poisson analysis were similar to the dichotomized results, which found that work stress, peer influence, and academic aspiration were predictors of alcohol use and drunkenness.

## Discussion

In the present study, we explored the association between work stress with alcohol use and drunkenness in a nationally-representative sample of high school seniors in the United States. Academic aspiration was negatively associated with alcohol use and drunkenness, while work stress and negative peer influence were positively associated with alcohol use and drunkenness in adolescents after controlling for confounders and demographic variables. These associations were supported by past research which found that educational attainment and peer effects predicted alcohol use in adolescents [27, 30, 34].

Adolescents with work stress (low job satisfaction, and dissatisfaction with perceived personal safety and the safety of their possessions) were more likely to use alcohol. This suggested that adolescents who experienced stress at work had underlying risk factors for problematic alcohol use. The perceived dissatisfaction at work could be caused by characteristics of the work environment such as unsafe work conditions, co-workers, or job roles and responsibilities.

We also found that the relationship between work stress and alcohol use or drunkenness was moderated by peer influence. Past evidence suggested that these relationships could exist, but never tested the potential interactions [27, 30]. Conversely, lower levels of negative peer influence buffered against potentially negative consequences of work stress for adolescents engaged in employment. Indeed, social support has been shown to buffer individuals from the negative effects of stress [37]. Positive peer influences should be encouraged in order delay the onset of alcohol use in adolescents. For example, adolescents with friends who work could discourage alcohol use in order to reduce the risk. Adolescents that experienced work stress and negative peer support may be more vulnerable to embracing adverse drinking habits. The influence of these variables had a multiplicative effect and adolescents that exposed to both were more likely to use alcohol.

Past research demonstrated that stressful life events were strongly associated with alcohol use disorders in adolescence [11, 38]. Stressful life events created the feeling of meaningless in one’s life, which was then treated by drug substances in adolescents. The relationship between stress and alcohol use could also be influenced by individual characteristics such as gender, coping mechanisms, and alcohol use expectations [16, 22, 38]. In this study, we also observed that individual perceptions towards alcohol use, as well as social influences played a role, which affected this relationship. Alcohol use was therefore an outcome that was associated with many variables, which were modified by social structures and individual characteristics.

Our study has some limitations. First, data was collected from cross-sectional surveys and thus temporality could not be determined. It was unclear whether work stress occurred before or after alcohol use because of the nature of the study design. Second, work stress was created based on the questions asked in the survey, including job satisfaction, perceived safety at work, and perceived safety of one’s possessions. While other studies measured stress by negative life events [12, 38], our definition of work stress should be a valid predictor based on similar definitions in another study [39]. Third, we did not measure the pattern of alcohol use or drunkenness over the lifetime, past 12 months, or past 30 days. It could be possible that alcohol use on multiple occasions was a result of one or many stressful work events, thus binge drinking may be more important to consider. Fourth, our study did not capture adolescents that dropped out of high school. It is likely that adolescents who drop out were more likely to be in lower SES families, more likely to use alcohol, and thus likely to work in high stress manual labor jobs. Thus, our results may not be generalizable to adolescents who did not attend high school. Fifth, due to large sample size, effect estimates of small magnitude achieve statistical significance but may have limited population health relevance. However, alcohol consumption among adolescents is widespread, then small effects can translate to meaningful population health findings, suggesting that the small magnitudes of some of our effect estimates remain potentially important at the population level. Future studies will need to establish the effect of work stress and alcohol use, as well as conduct longitudinal studies to measure temporality.

## Conclusion

The findings presented evidence that alcohol use among adolescents could be influenced by characteristics of the work environment. Our study presented job satisfaction, perceived personal safety at work, and perceived safety of possessions as contributors to work stress. The effect of work stress was exacerbated by high negative peer influences that encouraged adolescents to consume alcohol. Alternatively, positive peer influences could buffer the negative effect of work stress on alcohol use. These findings could have important implications on our understanding of workplace factors associated with alcohol use in adolescents. Specifically, working in an unsafe environment where the individual feels threatened or unsatisfied could be considered external factors that potentially influence alcohol use. Our findings supported past studies which demonstrated that peer influences contributed to alcohol use. Individual characteristics, work environment, and social support should be considered in future studies to understand how they interact with work stress to contribute to early onset of alcohol use in working adolescents.

## Consent

The Monitoring the Future study protocols regarding respondent consent have been approved by the Institutional Review Board of the University of Michigan.

## Electronic supplementary material

Additional file 1: **Supplementary Tables.** (DOCX 42 KB)

## Abbreviations

C.I.: Confidence interval
MTF: Monitoring the Future national survey
SAS: SAS statistical software.
