# Supplementary material for: Work stress and alcohol consumption among adolescents: moderation by family and peer influences
Source: BMC Public Health. 2014 Dec 18;14:1303. doi: 10.1186/1471-2458-14-1303 (PMC4301940; doi:10.1186/1471-2458-14-1303)
Supplement: Supplementary file 1 — Additional file 1: Supplementary Tables. (DOCX 42 KB) [file 12889_2014_7435_MOESM1_ESM.docx]

**Additional file 1**

**Supplement Table 1: Poisson Regression for Work Stress and Academic Aspiration on Alcohol use and Drunkenness**

Analysis of Parameter Estimates for Alcohol use over the lifetime

|  | Estimate | Standard Error | Wald chi-square | p-value |
| --- | --- | --- | --- | --- |
| No work stress (0)* | 0 | - | - | - |
| 1 | -0.011 | 0.026 | 0.18 | 0.673 |
| 2 | -0.054 | 0.041 | 1.76 | 0.184 |
| Highest level of work stress (3) | -0.019 | 0.096 | 0.04 | 0.841 |
| Lowest level of academic aspiration (0)* | 0 | - | - | - |
| 1 | -0.022 | 0.110 | 0.04 | 0.842 |
| 2 | -0.116 | 0.107 | 1.18 | 0.278 |
| 3 | -0.162 | 0.105 | 2.40 | 0.121 |
| 4 | -0.238 | 0.104 | 5.27 | 0.022 |
| 5 | -0.244 | 0.102 | 5.67 | 0.017 |
| 6 | -0.301 | 0.102 | 8.70 | 0.003 |
| 7 | -0.406 | 0.102 | 15.87 | <.0001 |
| Highest level of academic aspiration (8) | -0.599 | 0.104 | 33.52 | <.0001 |

*controlling for life satisfaction, GPA, gender, race, region, and parental education

Analysis of Parameter Estimates for Alcohol use over the past 12 months

|  | Estimate | Standard Error | Wald chi-square | p-value |
| --- | --- | --- | --- | --- |
| No work stress (0)* | 0 | - | - | - |
| 1 | 0.012 | 0.029 | 0.16 | 0.686 |
| 2 | -0.062 | 0.046 | 1.78 | 0.182 |
| Highest level of work stress (3) | -0.035 | 0.110 | 0.10 | 0.746 |
| Lowest level of academic aspiration (0)* | 0 | - | - | - |
| 1 | -0.127 | 0.117 | 1.18 | 0.278 |
| 2 | -0.248 | 0.114 | 4.74 | 0.029 |
| 3 | -0.337 | 0.112 | 9.06 | 0.003 |
| 4 | -0.362 | 0.110 | 10.80 | 0.001 |
| 5 | -0.413 | 0.109 | 14.39 | 0.0001 |
| 6 | -0.480 | 0.108 | 19.59 | <.0001 |
| 7 | -0.589 | 0.108 | 29.51 | <.0001 |
| Highest level of academic aspiration (8) | -0.808 | 0.111 | 53.49 | <.0001 |

*controlling for life satisfaction, GPA, gender, race, region, and parental education

Analysis of Parameter Estimates for Alcohol use over the past 30 days

|  | Estimate | Standard Error | Wald chi-square | p-value |
| --- | --- | --- | --- | --- |
| No work stress (0)* | 0 | - | - | - |
| 1 | -0.015 | 0.035 | 0.18 | 0.671 |
| 2 | -0.109 | 0.056 | 3.74 | 0.053 |
| Highest level of work stress (3) | 0.004 | 0.126 | 0.00 | 0.976 |
| Lowest level of academic aspiration (0)* | 0 | - | - | - |
| 1 | -0.227 | 0.126 | 3.23 | 0.072 |
| 2 | -0.386 | 0.123 | 9.84 | 0.002 |
| 3 | -0.519 | 0.121 | 18.42 | <.0001 |
| 4 | -0.625 | 0.119 | 27.43 | <.0001 |
| 5 | -0.604 | 0.117 | 26.54 | <.0001 |
| 6 | -0.701 | 0.117 | 36.14 | <.0001 |
| 7 | -0.903 | 0.117 | 59.44 | <.0001 |
| Highest level of academic aspiration (8) | -1.173 | 0.121 | 94.81 | <.0001 |

*controlling for life satisfaction, GPA, gender, race, region, and parental education

Analysis of Parameter Estimates for Drunkenness over the lifetime

|  | Estimate | Standard Error | Wald chi-square | p-value |
| --- | --- | --- | --- | --- |
| No work stress (0)* | 0 | - | - | - |
| 1 | -0.012 | 0.038 | 0.11 | 0.742 |
| 2 | -0.089 | 0.060 | 2.19 | 0.139 |
| Highest level of work stress (3) | -0.025 | 0.140 | 0.03 | 0.856 |
| Lowest level of academic aspiration (0)* | 0 | - | - | - |
| 1 | -0.101 | 0.151 | 0.45 | 0.503 |
| 2 | -0.185 | 0.147 | 1.59 | 0.207 |
| 3 | -0.299 | 0.144 | 4.32 | 0.038 |
| 4 | -0.354 | 0.142 | 6.23 | 0.013 |
| 5 | -0.329 | 0.140 | 5.51 | 0.019 |
| 6 | -0.447 | 0.140 | 10.29 | 0.001 |
| 7 | -0.597 | 0.140 | 18.26 | <.0001 |
| Highest level of academic aspiration (8) | -0.842 | 0.143 | 34.87 | <.0001 |

*controlling for life satisfaction, GPA, gender, race, region, and parental education

Analysis of Parameter Estimates for Drunkenness over the past 12 months

|  | Estimate | Standard Error | Wald chi-square | p-value |
| --- | --- | --- | --- | --- |
| No work stress (0)* | 0 | - | - | - |
| 1 | -0.035 | 0.051 | 0.46 | 0.499 |
| 2 | -0.149 | 0.082 | 3.29 | 0.070 |
| Highest level of work stress (3) | -0.147 | 0.189 | 0.61 | 0.436 |
| Lowest level of academic aspiration (0)* | 0 | - | - | - |
| 1 | -0.144 | 0.175 | 0.68 | 0.411 |
| 2 | -0.322 | 0.170 | 3.56 | 0.059 |
| 3 | -0.459 | 0.166 | 7.62 | 0.006 |
| 4 | -0.668 | 0.166 | 16.20 | <.0001 |
| 5 | -0.589 | 0.162 | 13.17 | 0.0003 |
| 6 | -0.769 | 0.162 | 22.62 | <.0001 |
| 7 | -1.069 | 0.163 | 43.02 | <.0001 |
| Highest level of academic aspiration (8) | -1.511 | 0.170 | 79.01 | <.0001 |

*controlling for life satisfaction, GPA, gender, race, region, and parental education

Analysis of Parameter Estimates for Drunkenness over the past 30 days

|  | Estimate | Standard Error | Wald chi-square | p-value |
| --- | --- | --- | --- | --- |
| No work stress (0)* | 0 | - | - | - |
| 1 | -0.005 | 0.079 | 0.00 | 0.955 |
| 2 | -0.204 | 0.133 | 2.36 | 0.124 |
| Highest level of work stress (3) | -0.067 | 0.273 | 0.06 | 0.807 |
| Lowest level of academic aspiration (0)* | 0 | - | - | - |
| 1 | -0.466 | 0.230 | 4.10 | 0.043 |
| 2 | -0.632 | 0.223 | 8.06 | 0.0005 |
| 3 | -0.918 | 0.219 | 17.67 | <.0001 |
| 4 | -1.092 | 0.215 | 25.85 | <.0001 |
| 5 | -1.171 | 0.212 | 30.58 | <.0001 |
| 6 | -1.311 | 0.209 | 39.17 | <.0001 |
| 7 | -1.839 | 0.215 | 73.34 | <.0001 |
| Highest level of academic aspiration (8) | -2.260 | 0.229 | 97.20 | <.0001 |

*controlling for life satisfaction, GPA, gender, race, region, and parental education

**Supplement Table 2: Poisson Regression for Work Stress and Peer Influence on Alcohol use and Drunkenness**

Analysis of Parameter Estimates for Alcohol use over the lifetime

|  | Estimate | Standard Error | Wald chi-square | p-value |
| --- | --- | --- | --- | --- |
| No work stress (0)* | 0 | - | - | - |
| 1 | 0.005 | 0.026 | 0.04 | 0.847 |
| 2 | -0.022 | 0.040 | 0.29 | 0.588 |
| Highest level of work stress (3) | 0.02 | 0.096 | 0.05 | 0.832 |
| Lowest level of negative peer influence (0)* | 0 | - | - | - |
| 1 | -0.011 | 0.279 | 0.00 | 0.969 |
| 2 | 0.355 | 0.271 | 1.71 | 0.191 |
| 3 | 0.526 | 0.269 | 3.82 | 0.051 |
| 4 | 0.864 | 0.268 | 10.36 | 0.001 |
| Highest level of negative peer influence (5) | 1.154 | 0.269 | 18.46 | <.0001 |

*controlling for life satisfaction, GPA, gender, race, region, and parental education

Analysis of Parameter Estimates for Alcohol use over the past 12 months

|  | Estimate | Standard Error | Wald chi-square | p-value |
| --- | --- | --- | --- | --- |
| No work stress (0)* | 0 | - | - | - |
| 1 | 0.030 | 0.029 | 1.05 | 0.306 |
| 2 | -0.022 | 0.046 | 0.23 | 0.630 |
| Highest level of work stress (3) | 0.014 | 0.109 | 0.02 | 0.900 |
| Lowest level of negative peer influence (0)* | 0 | - | - | - |
| 1 | -0.196 | 0.341 | 0.28 | 0.596 |
| 2 | 0.476 | 0.358 | 1.77 | 0.184 |
| 3 | 0.750 | 0.356 | 4.45 | 0.035 |
| 4 | 1.163 | 0.355 | 10.74 | 0.001 |
| Highest level of negative peer influence (5) | 1.510 | 0.355 | 18.12 | <.0001 |

*controlling for life satisfaction, GPA, gender, race, region, and parental education

Analysis of Parameter Estimates for Alcohol use over the past 30 days

|  | Estimate | Standard Error | Wald chi-square | p-value |
| --- | --- | --- | --- | --- |
| No work stress (0)* | 0 | - | - | - |
| 1 | 0.014 | 0.035 | 0.16 | 0.686 |
| 2 | -0.051 | 0.056 | 0.82 | 0.367 |
| Highest level of work stress (3) | 0.078 | 0.125 | 0.39 | 0.532 |
| Lowest level of negative peer influence (0)* | 0 | - | - | - |
| 1 | -0.669 | 0.440 | 2.31 | 0.128 |
| 2 | -0.079 | 0.417 | 0.04 | 0.851 |
| 3 | 0.350 | 0.411 | 0.72 | 0.395 |
| 4 | 1.070 | 0.410 | 6.82 | 0.009 |
| Highest level of negative peer influence (5) | 1.546 | 0.410 | 14.25 | 0.0002 |

*controlling for life satisfaction, GPA, gender, race, region, and parental education

Analysis of Parameter Estimates for Drunkenness over the lifetime

|  | Estimate | Standard Error | Wald chi-square | p-value |
| --- | --- | --- | --- | --- |
| No work stress (0)* | 0 | - | - | - |
| 1 | 0.008 | 0.037 | 0.05 | 0.832 |
| 2 | -0.060 | 0.060 | 1.02 | 0.312 |
| Highest level of work stress (3) | 0.007 | 0.139 | 0.00 | 0.963 |
| Lowest level of negative peer influence (0)* | 0 | - | - | - |
| 1 | 1.146 | 1.011 | 1.29 | 0.257 |
| 2 | 1.732 | 1.003 | 2.95 | 0.086 |
| 3 | 2.073 | 1.001 | 4.29 | 0.038 |
| 4 | 2.605 | 1.001 | 6.78 | 0.009 |
| Highest level of negative peer influence (5) | 3.019 | 1.001 | 9.11 | 0.003 |

*controlling for life satisfaction, GPA, gender, race, region, and parental education

Analysis of Parameter Estimates for Drunkenness over the past 12 months

|  | Estimate | Standard Error | Wald chi-square | p-value |
| --- | --- | --- | --- | --- |
| No work stress (0)* | 0 | - | - | - |
| 1 | 0.013 | 0.051 | 0.07 | 0.792 |
| 2 | -0.054 | 0.082 | 0.44 | 0.509 |
| Highest level of work stress (3) | -0.026 | 0.188 | 0.02 | 0.892 |
| Lowest level of negative peer influence (0)* | 0 | - | - | - |
| 1 | 19.158 | 0 | - | - |
| 2 | 19.878 | 0.353 | 3175.92 | <.0001 |
| 3 | 20.591 | 0.353 | 4009.66 | <.0001 |
| 4 | 21.755 | 0.318 | 4677.25 | <.0001 |
| Highest level of negative peer influence (5) | 22.436 | 0.318 | 4987.95 | <.0001 |

*controlling for life satisfaction, GPA, gender, race, region, and parental education

Analysis of Parameter Estimates for Drunkenness over the past 30 days

|  | Estimate | Standard Error | Wald chi-square | p-value |
| --- | --- | --- | --- | --- |
| No work stress (0)* | 0 | - | - | - |
| 1 | 0.067 | 0.079 | 0.72 | 0.397 |
| 2 | -0.111 | 0.133 | 0.70 | 0.403 |
| Highest level of work stress (3) | 0.186 | 0.272 | 0.47 | 0.494 |
| Lowest level of negative peer influence (0)* | 0 | - | - | - |
| 1 | 18.390 | 0 | - | - |
| 2 | 18.709 | 0.521 | 1289.31 | <.0001 |
| 3 | 19.250 | 0.468 | 1689.64 | <.0001 |
| 4 | 20.750 | 0.451 | 2120.90 | <.0001 |
| Highest level of negative peer influence (5) | 21.068 | 0.450 | 2309.55 | <.0001 |

*controlling for life satisfaction, GPA, gender, race, region, and parental education

**Supplement Table 3: Poisson Regression for Work Stress and Parent Influence on Alcohol use and Drunkenness**

Analysis of Parameter Estimates for Alcohol use over the lifetime

|  | Estimate | Standard Error | Wald chi-square | p-value |
| --- | --- | --- | --- | --- |
| No work stress (0)* | 0 | - | - | - |
| 1 | 0.019 | 0.026 | 0.55 | 0.460 |
| 2 | -0.008 | 0.040 | 0.04 | 0.840 |
| Highest level of work stress (3) | 0.052 | 0.096 | 0.29 | 0.591 |
| Lowest level of positive parent influence (0)* | 0 | - | - | - |
| 1 | -0.031 | 0.052 | 0.36 | 0.547 |
| Highest level of positive parent influence (2) | -0.072 | 0.049 | 2.21 | 0.137 |

*controlling for life satisfaction, GPA, gender, race, region, and parental education

Analysis of Parameter Estimates for Alcohol use over the past 12 months

|  | Estimate | Standard Error | Wald chi-square | p-value |
| --- | --- | --- | --- | --- |
| No work stress (0)* | 0 | - | - | - |
| 1 | 0.049 | 0.029 | 2.76 | 0.096 |
| 2 | -0.006 | 0.046 | 0.02 | 0.897 |
| Highest level of work stress (3) | 0.052 | 0.110 | 0.23 | 0.634 |
| Lowest level of positive parent influence (0)* | 0 | - | - | - |
| 1 | -0.041 | 0.059 | 0.49 | 0.485 |
| Highest level of positive parent influence (2) | -0.093 | 0.055 | 2.86 | 0.091 |

*controlling for life satisfaction, GPA, gender, race, region, and parental education

Analysis of Parameter Estimates for Alcohol use over the past 30 days

|  | Estimate | Standard Error | Wald chi-square | p-value |
| --- | --- | --- | --- | --- |
| No work stress (0)* | 0 | - | - | - |
| 1 | 0.039 | 0.035 | 1.21 | 0.272 |
| 2 | -0.024 | 0.056 | 0.19 | 0.664 |
| Highest level of work stress (3) | 0.148 | 0.125 | 1.39 | 0.239 |
| Lowest level of positive parent influence (0)* | 0 | - | - | - |
| 1 | -0.040 | 0.070 | 0.32 | 0.574 |
| Highest level of positive parent influence (2) | -0.079 | 0.066 | 1.44 | 0.230 |

*controlling for life satisfaction, GPA, gender, race, region, and parental education

Analysis of Parameter Estimates for Drunkenness over the lifetime

|  | Estimate | Standard Error | Wald chi-square | p-value |
| --- | --- | --- | --- | --- |
| No work stress (0)* | 0 | - | - | - |
| 1 | 0.029 | 0.038 | 0.60 | 0.439 |
| 2 | -0.030 | 0.060 | 0.25 | 0.620 |
| Highest level of work stress (3) | 0.065 | 0.140 | 0.22 | 0.639 |
| Lowest level of positive parent influence (0)* | 0 | - | - | - |
| 1 | -0.009 | 0.076 | 0.01 | 0.905 |
| Highest level of positive parent influence (2) | -0.074 | 0.071 | 1.09 | 0.297 |

*controlling for life satisfaction, GPA, gender, race, region, and parental education

Analysis of Parameter Estimates for Drunkenness over the past 12 months

|  | Estimate | Standard Error | Wald chi-square | p-value |
| --- | --- | --- | --- | --- |
| No work stress (0)* | 0 | - | - | - |
| 1 | 0.035 | 0.051 | 0.47 | 0.491 |
| 2 | -0.035 | 0.082 | 0.18 | 0.667 |
| Highest level of work stress (3) | 0.012 | 0.189 | 0.00 | 0.952 |
| Lowest level of positive parent influence (0)* | 0 | - | - | - |
| 1 | -0.082 | 0.096 | 0.74 | 0.390 |
| Highest level of positive parent influence (2) | -0.213 | 0.090 | 5.65 | 0.018 |

*controlling for life satisfaction, GPA, gender, race, region, and parental education

Analysis of Parameter Estimates for Drunkenness over the past 30 days

|  | Estimate | Standard Error | Wald chi-square | p-value |
| --- | --- | --- | --- | --- |
| No work stress (0)* | 0 | - | - | - |
| 1 | 0.095 | 0.079 | 1.44 | 0.230 |
| 2 | -0.054 | 0.133 | 0.16 | 0.686 |
| Highest level of work stress (3) | 0.236 | 0.272 | 0.75 | 0.385 |
| Lowest level of positive parent influence (0)* | 0 | - | - | - |
| 1 | 0.011 | 0.160 | 0.00 | 0.946 |
| Highest level of positive parent influence (2) | -0.0140 | 0.151 | 0.86 | 0.353 |

*controlling for life satisfaction, GPA, gender, race, region, and parental education

**Supplemental Table 4: Odds ratios of alcohol use and drunkenness in adolescents who worked**

| Hours Worked every week* | Alcohol use | | | Drunkenness |  |  |
| --- | --- | --- | --- | --- | --- | --- |
|  | Lifetime | 12 months | 30 days | Lifetime | 12 months | 30 days |
| 0 (reference) |  |  |  |  |  |  |
| 5 or less | 1.058 (0.99-1.13) | 1.08 (1.01-1.16) | 1.11 (1.04-1.20) | 1.05 (0.89-1.24) | 1.19 (0.98-1.44) | 1.22 (0.93-1.61) |
| 6-10 | 1.28 (1.2-1.367) | 1.30 (1.21-1.39) | 1.38 (1.29-1.48) | 1.45 (1.23-1.70) | 1.48 (1.23-1.78) | 1.40 (1.08-1.80) |
| 11-15 | 1.50 (1.41-1.60) | 1.52 (1.42-1.62) | 1.49 (1.39-1.59) | 1.77 (1.51-2.08) | 1.81 (1.52-2.17) | 1.62 (1.27-2.07) |
| 16-20 | 1.61 (1.52-1.71) | 1.64 (1.55-1.75) | 1.60 (1.50-1.70) | 1.92 (1.66-2.23) | 1.98 (1.68-2.34) | 1.76 (1.40-2.21) |
| 21-25 | 1.83 (1.71-1.95) | 1.83 (1.71-1.96) | 1.70 (1.59-1.82) | 1.89 (1.60-2.23) | 2.28 (1.90-2.74) | 2.56 (2.03-3.22) |
| 26-30 | 1.90 (1.76-2.06) | 1.89 (1.54-2.04) | 1.75 (1.61-1.89) | 2.16 (1.77-2.65) | 2.01 (1.62-2.51) | 2.27 (1.72-2.98) |
| 30+ | 2.07 (1.91-2.24) | 1.98 (1.82-2.14) | 1.94 (1.79-2.10) | 1.82 (1.48-2.24) | 2.02 (1.61-2.53) | 1.93 (1.45-2.58) |

*reference group is 0 hours worked per week

**Supplemental Table 5: Odds ratios of alcohol use and drunkenness among adolescents who earned adolescents**

| Money earned ($)* | Alcohol use | | | Drunkenness |  |  |
| --- | --- | --- | --- | --- | --- | --- |
|  | Lifetime | 12 months | 30 days | Lifetime | 12 months | 30 days |
| 0 (reference) |  |  |  |  |  |  |
| 1-5 | 0.99 (0.80-1.22) | 0.99 (0.79-1.23) | 1.09 (0.87-1.37) | 1.75 (1.06-2.88) | 0.92 (0.48-1.76) | 0.64 (0.23-1.80) |
| 6-10 | 1.12 (1.01-1.25) | 1.11 (1.00-1.25) | 1.19 (1.06-1.33) | 1.34 (1.03-1.73) | 1.44 (1.07-1.93) | 1.50 (1.01-2.24) |
| 11-20 | 1.09 (0.97-1.22) | 1.09 (0.97-1.23) | 1.22 (1.08-1.38) | 1.44 (1.07-1.93) | 1.70 (1.23-2.36) | 1.89 (1.25-2.86) |
| 21-35 | 1.14 (1.03-1.26) | 1.16 (1.05-1.28) | 1.17 (1.05-1.29) | 1.23 (0.97-1.56) | 1.54 (1.18-2.01) | 1.38 (0.94-2.03) |
| 36-50 | 1.38 (1.27-1.50) | 1.36 (1.25-1.49) | 1.42 (1.31-1.55) | 1.46 (1.18-1.79) | 1.50 (1.18-1.91) | 1.53 (1.11-2.12) |
| 51-75 | 1.51 (1.41-1.61) | 1.58 (1.47-1.69) | 1.52 (1.41-1.63) | 1.63 (1.37-1.93) | 1.83 (1.50-2.22) | 1.62 (1.24-2.12) |
| 76-125 | 1.66 (1.57-1.75) | 1.69 (1.60-1.79) | 1.62 (1.54-1.72) | 1.95 (1.71-2.23) | 2.15 (1.85-2.49) | 1.84 (1.50-2.25) |
| 126-175 | 2.01 (1.91-2.12) | 1.99 (1.89-2.11) | 1.93 (1.82-2.03) | 2.11 (1.85-2.41) | 2.25 (1.94-2.62) | 2.35 (1.93-2.85) |
| 176+ | 2.29 (1.98-2.64) | 2.41 (2.08-2.79) | 2.32 (2.01-2.67) | 2.56 (1.76-3.72) | 2.97 (2.00-4.40) | 2.39 (1.47-3.89) |

*reference group is 0 money earned

**Supplemental Table 6: Odds ratios of alcohol use and drunkenness among adolescents with increasing levels of work stress**

| Levels of  Work Stress* (% of total) | Alcohol use | | | Drunkenness |  |  |
| --- | --- | --- | --- | --- | --- | --- |
|  | lifetime | 12 months | 30 days | lifetime | 12 months | 30 days |
| Reference (66.29) |  |  |  |  |  |  |
| 1 (23.22) | 1.05 (0.95-1.17) | 1.16 (1.04-1.29) | 1.08 (0.97-1.20) | 1.10 (0.98-1.23) | 1.09 (0.96-1.24) | 1.15 (0.97-1.36) |
| 2 (8.95) | 0.99 (0.84-1.17) | 1.01 (0.85-1.19) | 0.97 (0.82-1.14) | 0.98 (0.82-1.17) | 1.00 (0.82-1.23) | 0.98 (0.74-1.30) |
| 3 (1.54) | 1.23 (0.83-1.82) | 1.20 (0.80-1.79) | 1.31 (0.89-1.93) | 1.27 (0.81-1.98) | 1.13 (0.70-1.83) | 1.45 (0.80-2.64) |

*reference group was adolescents with no work stress (i.e., they were satisfied with their job, personal safety at work, and safety of possessions at work)

**Supplemental Table 7: Odds ratios of alcohol use and drunkenness among adolescents with increasing levels of academic aspiration**

| Levels of academic aspiration (% of total) | Alcohol use | | | Drunkenness |  |  |
| --- | --- | --- | --- | --- | --- | --- |
|  | lifetime | 12 months | 30 days | lifetime | 12 months | 30 days |
| 0 (2.20) | - | - | - | - | - | - |
| 1 (10.36) | 0.85 (0.48-1.51) | 0.56 (0.33-0.96) | 0.51 (0.30-0.85) | 0.47 (0.18-1.27) | 0.58 (0.26-1.30) | 0.42 (0.22-0.82) |
| 2 (5.14) | 0.58 (0.33-1.00) | 0.37 (0.22-0.62) | 0.35 (0.21-0.58) | 0.30 (0.12-0.78) | 0.33 (0.15-0.71) | 0.33 (0.17-0.62) |
| 3 (7.38) | 0.48 (0.28-0.82) | 0.27 (0.16-0.46) | 0.27 (0.16-0.43) | 0.20 (0.08-0.50) | 0.24 (0.11-0.51) | 0.22 (0.12-0.42) |
| 4 (9.68) | 0.36 (0.21-0.61) | 0.25 (0.15-0.42) | 0.22 (0.13-0.35) | 0.17 (0.07-0.43) | 0.16 (0.08-0.34) | 0.18 (0.10-0.33) |
| 5 (13.03) | 0.35 (0.21-0.59) | 0.22 (0.13-0.36) | 0.23 (0.14-0.37) | 0.18 (0.07-0.46) | 0.19 (0.09-0.39) | 0.16 (0.09-0.30) |
| 6 (16.67) | 0.30 (0.18-0.50) | 0.18 (0.11-0.30) | 0.19 (0.12-0.31) | 0.13 (0.05-0.33) | 0.14 (0.07-0.29) | 0.14 (0.07-0.25) |
| 7 (20.03) | 0.22 (0.13-0.37) | 0.14 (0.08-0.23) | 0.14 (0.09-0.23) | 0.10 (0.04-0.24) | 0.09 (0.04-0.18) | 0.07 (0.04-0.14) |
| 8 (15.51) | 0.14 (0.08-0.23) | 0.08 (0.05-0.14) | 0.09 (0.06-0.15) | 0.06 (0.02-0.16) | 0.05 (0.02-0.10) | 0.05 (0.03-0.09) |

*reference group was 0 (lowest level of academic aspiration)

**Supplemental Table 8: Odds ratios of alcohol use and drunkenness among adolescents with increasing levels of positive parent influence**

| Levels of positive parent influence (% of total) | Alcohol use | | | Drunkenness |  |  |
| --- | --- | --- | --- | --- | --- | --- |
|  | lifetime | 12 months | 30 days | lifetime | 12 months | 30 days |
| 0 (5.24) | - | - | - | - | - | - |
| 1 (21.43) | 0.85 (0.69-1.05) | 0.89 (0.71-1.10) | 0.93 (0.75-1.15) | 0.96 (0.76-1.23) | 0.85 (0.66-1.10) | 1.02 (0.72-1.44) |
| 2 (73.33) | 0.75 (0.62-0.92) | 0.77 (0.62-0.94) | 0.86 (0.70-1.05) | 0.83 (0.66-1.04) | 0.69 (0.54-0.88) | 0.84 (0.61-1.17) |

*reference group was 0 (lowest level of positive parent influence)

**Supplemental Table 9: Odds ratios of alcohol use and drunkenness among adolescents with increasing levels of negative peer influence**

| Levels of negative peer influence (% of total) | Alcohol use | | | Drunkenness |  |  |
| --- | --- | --- | --- | --- | --- | --- |
|  | lifetime | 12 months | 30 days | lifetime | 12 months | 30 days |
| 0 (1.18) | - | - | - | - | - | - |
| 1 (8.51) | 0.96 (0.44-2.08) | 0.80 (0.33-1.98) | 0.54 (0.18-1.65) | 3.34 (0.44-25.47) | >999.99 | >999.99 |
| 2 (12.94) | 1.95 (0.91-4.14) | 2.24 (0.93-5.39) | 1.17 (0.40-3.40) | 6.96 (0.93-52.26) | >999.99 | >999.99 |
| 3 (21.44) | 2.86 (1.35-6.04) | 3.77 (1.58-9.01) | 1.99 (0.69-5.73) | 11.38 (1.52-85.106) | >999.99 | >999.99 |
| 4 (33.33) | 8.00 (3.78-16.90) | 11.34 (4.75-27.04) | 6.02 (2.09-17.31) | 33.16 (4.44-247.82) | >999.99 | >999.99 |
| 5 (22.60) | 25.41 (11.98-53.89) | 42.64 (17.81-102.08) | 17.75 (6.16-51.12) | 224.07 (29.85->999.99) | >999.99 | >999.99 |

*reference group was 0 (lowest level of negative peer influence)
